# Cancer Diagnosis Using a Liquid Biopsy: Challenges and Expectations

**DOI:** 10.3390/diagnostics8020031

**Published:** 2018-05-09

**Authors:** Francesc Castro-Giner, Sofia Gkountela, Cinzia Donato, Ilaria Alborelli, Luca Quagliata, Charlotte K. Y. Ng, Salvatore Piscuoglio, Nicola Aceto

**Affiliations:** 1 Cancer Metastasis Laboratory, Department of Biomedicine, University of Basel and University Hospital Basel, 4058 Basel, Switzerland; francesc.castro@unibas.ch (F.C.-G.); sofia.gkountela@unibas.ch (S.G.); cinzia.donato@unibas.ch (C.D.); 2 Swiss Institute of Bioinformatics, 1015 Lausanne, Switzerland; 3 Institute of Pathology, University Hospital Basel, 4031 Basel, Switzerland; ilaria.alborelli@usb.ch (I.A.); Luca.Quagliata@usb.ch (L.Q.); kiuyancharlotte.ng@usb.ch (C.K.Y.N.); Salvatore.Piscuoglio@usb.ch (S.P.); 4 Hepatology Laboratory, Department of Biomedicine, University of Basel and University Hospital Basel, 4031 Basel, Switzerland

**Keywords:** circulating tumor cells, single cell analysis, deep-sequencing, liquid biopsy, cancer diagnosis, cancer treatment

## Abstract

The field of cancer diagnostics has recently been impacted by new and exciting developments in the area of liquid biopsy. A liquid biopsy is a minimally invasive alternative to surgical biopsies of solid tissues, typically achieved through the withdrawal of a blood sample or other body fluids, allowing the interrogation of tumor-derived material including circulating tumor cells (CTCs) and circulating tumor DNA (ctDNA) fragments that are present at a given time point. In this short review, we discuss a few studies that summarize the state-of-the-art in the liquid biopsy field from a diagnostic perspective, and speculate on current challenges and expectations of implementing liquid biopsy testing for cancer diagnosis and monitoring in the clinical setting.

## 1. Background

The metastatic spread of cancer to distant sites remains the main cause of death for cancer patients in ~90% of the cases [1,2,3]. During tumor manifestation and progression, treatment decisions are generally made based on the results of diagnostic routine testing of primary tumor specimens. However, evolutionary changes in the genetic and epigenetic landscape of tumors have been shown to occur [4,5,6,7], and present a major challenge for treatment selection. Particularly, cancers appear to undergo Darwinian evolutionary principles as a function of time, influenced by genetic and epigenetic variability, clonal selection as a consequence to external pressure (e.g., drug treatment), microenvironmental stimuli (e.g., the immune system), as well as other factors (e.g., the concept of neutral evolution) [8,9]. As a result, to increase its fitness, a progressing cancer features heterogeneity and constant evolution, posing a significant challenge to its eradication. As a further confounding factor, tumor biopsies typically achieve the sampling of only a part of the tumor and thus may only capture a fraction of this heterogeneity, hence not being fully informative about the levels of genetic and epigenetic variability of a patient’s cancer. While it is unlikely for a patient to undergo serial biopsies of primary and metastatic lesions along tumor progression, liquid biopsy represents a minimally invasive and more sustainable alternative to interrogate cancer cells longitudinally.

Liquid biopsy is a term that refers to the sampling of non-solid biological tissue, most commonly of blood, but also saliva, urine, cerebrospinal fluid and other body fluids. Liquid biopsy from cancer patients is mainly used for the extraction of circulating tumor cells (CTCs), circulating tumor DNA (ctDNA), and eventually other tumor-derived material (e.g., exosomes) [10,11]. CTCs are shed in the bloodstream from primary and metastatic lesions, yet they are highly diluted among billions of blood cells, even in patients with advanced metastatic disease (0–10 CTCs per mL of blood) [12]. Nevertheless, CTC detection has been possible through the development of specialized technologies that are based on various principles, including antibody capture [13,14,15,16], size exclusion [17], depletion of red and white blood cells [18], or dielectrophoresis [19], among others. Through these technologies, CTCs have been detected in virtually all cancer types [20], and their abundance has been shown to correlate with disease aggressiveness [21,22,23,24,25,26,27,28,29,30]. Morphologically, CTCs are present as single cells or clusters of cells (CTC clusters), with the latter being associated with a higher metastatic potential [31]. In terms of output, CTC extraction from a blood sample allows the investigation of a number of cancer-associated phenomena, including but not limited to the presence of mutations, translocations, genetic rearrangements, loss of heterozygosity, gene overexpression and downregulation, alternative splicing, protein expression and drug sensitivity. In contrast, ctDNA is derived from tumor cells undergoing apoptosis or necrosis, and it represents a fraction of the total cell-free DNA (cfDNA) that is circulating in the blood of patients and that is derived from physiological tissue remodeling events [32]. Generally, ctDNA represents between <0.1–10% of the total cfDNA detectable in human blood [33], a quantity that may vary as a consequence of tumor burden, inflammatory status, cellular turnover and accessibility of cancer cells to blood vessels [33]. Interestingly, cancer patients have much higher levels of total cfDNA than healthy individuals [34]. When tumors increase in volume, so does the cellular turnover and hence the number of apoptotic and necrotic cells within the tumor tissue itself, leading to the release of ctDNA into the bloodstream, admixed with normal cfDNA. Most DNA fragments in circulation, including cfDNA and ctDNA, measure between 180 and 200 nucleotides in size, suggesting that apoptosis is likely to produce the majority of DNA fragments found in the bloodstream. Interestingly, shorter ctDNA fragments have been reported in at least some tumor types (e.g., hepatocellular carcinoma) as well as large cfDNA fragments of thousands of base pairs, which are probably the result of tissue necrosis [35]. A typical output for ctDNA analysis is the assessment of genetic alteration events in well-established cancer-associated genes, made possible through the advent of next-generation sequencing (NGS) technologies that allow the detection of cancer-derived ctDNA sequences that are greatly diluted among normal cfDNA in patients [36]. Additionally to CTCs and ctDNA, exosomes have recently received increased attention as they also represent tumor-derived material that can be found in a variety of body fluids (including the blood) of patients [11]. Exosomes contain proteins, RNAs and DNA from a patient’s tumor and provide an additional tool to investigate cancer-related features in real time. Techniques for exosome interrogation and their role in diagnosis, treatment and resistance to therapy are reviewed elsewhere [11].

Together, new technologies are enabling the interrogation of tumor-derived material from a liquid biopsy, allowing the achievement of longitudinal patient monitoring. In this short review, we discuss a few recent reports that support a role for CTCs and ctDNA analysis as tools for the monitoring and diagnosis of cancer. We also speculate on the advantages and disadvantages of liquid biopsy as opposed to a tissue biopsy, and discuss practical aspects of liquid biopsy implementation in the clinical setting, including challenges that are typically met during data analysis.

## 2. Circulating Tumor Cells as a Diagnostic Tool

CTCs represent cancer cells that have detached from a primary tumor or a metastatic lesion and are on their way to establish a metastatic lesion at a distant site. CTC enumeration in patients has provided an additional tool to determine disease aggressiveness and response to therapy, mostly through the Food and Drug Administration (FDA)-cleared CellSearch system, relying on the expression of EpCAM and Cytokeratin on cancer cells in circulation [13]. Beyond enumeration, the major challenge in using CTCs for diagnostic purposes has been the low number of CTCs that are typically isolated from patients, especially at disease onset [37], and difficulties in proceeding with single cell-resolution molecular analysis. However, with the development of technologies that are able to deal with minimal cell numbers, such as microfluidics and NGS [38], the liquid biopsy field is now in a position to address highly relevant questions.

For example, metastatic disease may differ in terms of key biomarkers when compared to the primary tumor. Phenotypic analysis of CTCs from patients diagnosed with estrogen receptor (ER)-positive/HER2-negative breast cancer (through histological assessment of the primary tumor), highlighted that CTCs are capable of acquiring HER2 expression in the metastatic setting [39]. Using a combination of CTC-culturing, single-cell RNA sequencing (scRNA-seq) and a small-scale drug screen combined with mass spectrometry analysis, it was shown that HER2-positive CTCs are more proliferative but not addicted to HER2 itself, while HER2-negative CTCs display the activation of Notch and DNA damage pathways, as well as resistance to cytotoxic therapy [39]. Combination treatment with paclitaxel and Notch inhibitors was sufficient to suppress the tumorigenic potential of both HER2-negative and HER2-positive phenotypes [39]. In a separate study, the RNA expression profile of CTCs from 50 patients with metastatic breast cancer revealed discrepancies between ER and HER2 levels of CTCs compared to matched primary tumors [40]. These studies, together with a number of other studies that were conducted comparing surgical biopsies of metastasis to the matched primary tumor [41,42,43], highlight that the commonly used biomarkers for treatment decisions tend to dynamically change during the course of the disease, and that monitoring patients longitudinally may enable more timely treatment decisions based on individual cases.

CTC analysis has also proven useful to assess the likelihood of a patient to respond to a given therapy before the therapy is administered (i.e., patient stratification). In small-cell lung cancer, copy number variations (CNVs) were determined in CTCs prior to chemotherapy, and allowed the identification of a CNV-classifier to determine chemosensitive versus chemorefractory individuals [44]. This CTC-based CNV classifier was able to correctly assign 83% of tested patients as chemosensitive or chemorefractory using pretreatment blood specimens. Of note, patients that were initially chemosensitive but then developed resistance did not acquire the same CNV profile associated with chemorefractory patients, indicating that the genetic basis of innate and acquired resistance may differ [44]. In a separate study, melanoma CTCs were profiled for RNA expression prior to treatment with immune checkpoint inhibitors [45]. This allowed the identification of a 19-gene digital RNA signature (CTC score) that prospectively correlated with improved progression-free survival and overall survival during therapy with immune checkpoint inhibitors in 49 patients [45]. Together, these studies represent examples of how CTC analysis can be used to noninvasively stratify patients prior to therapy initiation.

Further, CTCs have the potential to aid treatment decisions in the metastatic setting. This was elegantly shown for breast cancer (as well as other cancers), where CTCs could be propagated ex vivo under hypoxic conditions using a combination of growth factors and low attachment plates for more than six months [46,47,48]. In the context of personalized medicine, the achievement of CTC cultures carries the outstanding potential to noninvasively monitor the changing patterns of drug susceptibility in individual patients as their tumors acquire new mutations.

Taken together, these studies represent proof-of-concept studies of how CTC analysis can be used for clinical management and therapeutic purposes. Additionally to NGS, a number of well-established and accessible cell/molecular biology techniques can be applied to CTCs (e.g., immunostaining and fluorescence in situ hybridization) to detect specific biomarkers in the diagnostic setting [49]. However, molecular analysis of CTCs through NGS, especially when constrained by a low cell number, is still subject to challenges that may affect data interpretation. A description of these challenges is provided below.

## 3. Computational Challenges Associated with CTC Analysis

The development of single-cell sequencing technologies permits the interrogation of the transcriptome, genome, methylome and proteome of individual cells. The application of these technologies to CTC analysis has already shown a potential for cancer diagnosis, treatment selection, drug screening and disease monitoring [50,51,52,53]. Despite the improvements in single-cell sequencing protocols, strong stochastic variation, low coverage, high error rate and cell-to-cell heterogeneity are the major analytical challenges that persist and need to be overcome for proper data interpretation [54]. To overcome these challenges, computational methods have been developed that maximize the quality of single-cell data.

Gene expression analysis using RNA sequencing is the most established application for single-cell analysis. In contrast to bulk RNA sequencing, the gene expression signal from a single cell is characterized by a larger proportion of dropouts and a higher degree of noise. The high level of technical variability is caused by the lower sensitivity to detect lowly-expressed transcripts [55], typically determined by the efficiency of the cDNA synthesis step as well as the bias introduced during the amplification of the limited input material (whole transcriptome amplification, WTA) [56]. Sensitivity and technical variability can be quantified by adding a mix of polyadenylated mRNA molecules of known concentration (‘spike-ins’) [57]. In order to reduce the amplification bias, recent protocols also integrate unique molecular identifiers (UMIs) that tag each original mRNA molecule with a unique barcode [58,59].

Data preprocessing and quality control to remove biases from raw signals present a significant challenge for scRNA-seq analysis. Computational methods for RNA sequencing analysis developed for bulk sequencing are generally considered to be poorly applicable to single-cells, given the typical high levels of technical noise and gene dropouts. Different tools and workflows have been developed in order to detect poor quality libraries and to remove unwanted technical (e.g., batch effects) and biological (e.g., cell-cycle) variation [60,61,62,63]. Downstream analyses are then performed on normalized levels of expression, where normalization is a critical step that also accounts for read depth and capture efficiency [64,65,66]. These downstream analyses include the unsupervised clustering of cells based on their gene expression levels to detect cellular subpopulations and/or intermediate states, differential expression [64,65,67] and inference of gene regulatory networks [68,69,70,71,72,73,74,75,76]. Recently, however, a benchmarking study suggested that bulk RNA-seq analysis methods may perform similarly to methods specifically developed for scRNA-seq [77]. As there is no consensus yet, additional studies will be needed to define a standardized method for scRNA-seq data analysis.

The accurate detection of mutations from single-cells is quite challenging as it requires an intensive whole genome amplification (WGA). Typically, this results in non-uniform genome coverage, a high proportion of false-negatives due to low coverage breadth (50–95%) and allelic dropouts (10–50%), high false-positive rate due to errors introduced during the PCR amplification (≤10^−5^), and allelic imbalance [54,78]. Similar to scRNA-Seq, data processing for single cell DNA sequencing (scDNA-seq) relies on adopted computational methods originally developed for bulk sequencing, estimating the single cell error rate relative to bulk sequencing or control samples [51,54,79,80]. Currently, both single nucleotide variants (SNV) and copy number variants (CNVs) can be detected accurately. For SNV, the different methods that were recently developed overcome the inherent artifacts of scDNA-seq by either combining information across all the cells [81] or by modeling amplification biases through germline polymorphisms [82]. On the other hand, regarding CNV detection through scDNA-seq, current methods are generally limited to detecting large CNVs (megabase-scale) [83,84,85]. The accuracy can be partially improved by using protocols that produce more uniform coverage [54]. Among these, DNA amplification methods such as degenerate oligonucleotide primed PCR, multiple displacement amplification, multiple annealing and looping-based amplification cycles, as well as PicoPLEX, represent a few alternatives, with specific advantages and disadvantages in terms of coverage uniformity (reviewed elsewhere [54]). Alternatively, protocols based on single cell array comparative genomic hybridization (sc-aCGH) represent a rapid and cost-effective alternative for CNV interrogation, with some workflows allowing the detection of alterations with a resolution of 100 kb [86,87]. In addition, CNV could also be inferred from scRNA-seq by averaging the relative expression levels of genes over large genomic regions [68,69]. In principle, the mutational heterogeneity or the primary or metastatic tumor can be estimated through analysis of the mutational profile of CTCs, particularly in those patients with the highest numbers of detectable CTCs. To this end, probabilistic approaches were developed in order to overcome the high technical variation of single cell data and to enable the reconstruction of cell phylogenies using SNVs [88,89,90,91,92,93]. Some of these models are of particular interest for CTC analysis, because they address cell-doublets, and hence are appropriate for the analysis of CTC clusters [89,93].

Analyses of NGS data from CTCs present additional specific challenges. First, the extremely low numbers of CTCs typically extracted from a patient (e.g., between 0 and 10) [94] limit the use of current analytical approaches that, even though they are designed for single cells, require a large number of individual cells to be analyzed. As an alternative, ex vivo amplification of single cells in cell cultures could be used to expand the genomic material, at the expense of low efficiency (i.e., a low take rate of CTCs in culture) and the introduction of potential biases during the culturing process [46,95]. Second, CTCs are also found as clusters of cells of variable size [31]. If not properly controlled for, this might introduce systematic variability in the amplification process. In addition, extrinsic factors such as shear stress, immune attack and therapy can induce apoptosis [96,97], and hence modify the gene expression profile and reduce the quality of the extracted material. As an alternative, low quality cells can either be discarded before sequencing by running a low-pass sequencing [51] or after sequencing using extensive quality control. Finally, CTC signals can be masked by admixture with leukocytes, stromal cells and platelets [98], yet using gene expression signatures related to these cells it is possible to identify and eventually discard those samples with a high degree of admixture.

Recent technologies enable a more comprehensive characterization of single cells by performing parallel profiling of genome, transcriptome and methylome [99,100,101,102]. To our knowledge, there are no specific computational models designed to integrate data from different molecular layers and, at the same time, accommodate single-cell noise. The development of tailored computational methods for multi-omics studies will be critical for the future of single-cell analysis.

## 4. Circulating Tumor DNA as a Diagnostic Tool

Plasma-derived ctDNA is the most commonly used type of blood-based biomarker candidate in clinical practice, currently exceeding the use of CTCs. Thus far, the best-described use of ctDNA is for disease monitoring in cancer patients, to overcome the inherent risks associated with repeated tissue biopsies [34,103]. Specifically, ctDNA analysis has been mostly used to monitor the response to therapy, to detect minimal residual disease along treatment and to assess the development of therapy resistance [104,105,106,107,108,109]. The identification of resistance-associated genetic alterations found in ctDNA is now beginning to form the basis for treatment decision-making [110,111,112,113,114]. In fact, the FDA recently approved the first ctDNA test for *EGFR* mutations in non-small-cell lung cancer (cobas^®^ Mutation Test v2), a first indication of the imminent implementation of ctDNA testing in the clinical setting [115].

The potential role of ctDNA in cancer diagnosis is two-fold, namely, as a molecular surrogate for tissue biopsy at presentation and as a screening tool for early detection. The association between the quantity and fraction of ctDNA with tumor stage suggests the utility of ctDNA as a tissue surrogate in patients with a high disease burden [116,117]. ctDNA as a tissue surrogate is especially promising in poorly accessible tumors [118] and in tumors not routinely biopsied or excised, such as advanced hepatocellular carcinoma [119]. The presence of heterogeneity between the primary tumor and metastasis [5,120] also presents practical challenges in adequate tissue sampling and may be overcome by ctDNA profiling [121], although this will need to be further assessed in large patient cohorts.

The early detection of cancer in a screening setting may be an important approach to reduce cancer mortality. Of the many potential clinical utilities of ctDNA, early detection is arguably the most ambitious [122], as very large case–control cohorts are required to establish the sensitivity and specificity required to accurately identify patients with early-stage disease. Several studies have reported the detection of (actionable) genetic alterations in patients with early-stage disease [116,123,124,125,126]. However, patients with early-stage disease may harbor <1 mutant template molecules per milliliter of plasma [116,124], which is beyond the limit of detection of conventional NGS [127,128]. Large numbers of healthy control individuals will also be required to define the specificity of ctDNA tests before they can be considered for early cancer detection [128]. For instance, the combinatorial approach testing for *KRAS* mutations with four protein markers in the plasma to detect pancreatic cancer had superior specificity, albeit suboptimal sensitivity, and it may represent an alternative approach to using ctDNA for early detection [124]. Extending this combinatorial approach to the multi-analyte blood test CancerSEEK that detects eight common cancer types by assessing circulating proteins and mutations in cell-free DNA, the authors demonstrated a sensitivity of 69–95% (depending on cancer type) and a specificity of 99%, providing one of the first large-scale assessments of the potential of liquid biopsy that included >1000 patients and 850 healthy controls [125]. Clearly, there is also a question of whether the residual false positive cases may be a result of the presence of age-related clonal hematopoiesis in asymptomatic patients [126], particularly in those genes that were previously implicated in hematological cancers [129,130].

Together, these examples summarize the extraordinary potential of ctDNA for cancer diagnosis and screening, and the need to continue assessing sensitivity and specificity in large patient cohorts. It is however also important to mention that ctDNA derives from those cancer cells that died, and not from those that are actively proliferating [131], thus it is still unclear whether or not key alterations that are responsible for tumor progression can reliably be found from ctDNA analysis. Similarly to CTCs, however, NGS data analysis for ctDNA assessment presents several challenges that need to be taken into account for proper scoring, some of which are summarized below.

## 5. Computational Challenges Associated with ctDNA Analysis

In the context of cancer diagnosis and screening, accuracy and in particular specificity are of critical importance [122]. While highly sensitive techniques such as digital PCR (dPCR) or digital droplet PCR (ddPCR) can be used to detect somatic mutations in ctDNA at high sensitivity, they are limited to assaying one or a very small number of genomic loci, and the specific mutation to be assayed is typically determined a priori. On the other hand, NGS also allows the profiling of a much larger pool of genomic regions without having to predefine the specific alterations. However, the potential diagnostic utility of ctDNA is still largely determined by the signal-to-noise ratio of the assay. With conventional NGS, the limit of detection is around 1% (i.e., 1 out of 100 reads need to contain the given alteration). The nature of ctDNA makes it intrinsically rare, and its signal is diluted by other cfDNA derived from normal apoptotic cells, while it may overlap with the range of variant allele fractions due to technical artifacts. Technically, so-called “noise” in NGS may result from a large number of steps, including but not limited to DNA damage induced by extraction or sample fixation procedures, 8-oxo-dG, PCR errors/chimeras introduced during shearing in sequencing library preparation, base incorporation or homopolymer errors and imperfect imaging during sequence data acquisition, and incorrect alignment to the genome [132,133,134,135]. Besides biochemical repair, such as using uracil-DNA glycosylase (UDG) to minimize formalin fixation artifacts [136], various computational approaches can also partially reduce such artifacts.

Some of the errors described above, such as sequencing errors, are somewhat systematic and may be reduced by optimizing filtering strategies to distinguish likely true variants from background noise. For instance, spontaneous DNA deamination preferentially produces C > T/G > A substitutions while DNA oxidation leads to artifactual C > A/G > T substitutions [132,133,135]. The use of longer reads, although in this case limited to the length of ctDNA molecules, improves mapping accuracy. Other filtering strategies include filtering out variants identified in only one read in overlapping read pairs, with strong strand bias, or with low base quality scores. A better understanding of the error profiles has led to bioinformatic methods to optimize error correction [137,138,139]. Furthermore, aside from accounting for germline variants, the use of matched and/or a panel of germline samples also serves as an empirical filter to remove systematic sequencing or alignment artifacts [140]. The combination of biochemical and bioinformatic optimization can improve the limit of detection from 1% to up to 0.1% [137,138]. Of critical importance is the fact that spontaneous DNA deamination and oxidation are also naturally occurring in the aging process and contribute to tumor development [129,130] and should be carefully considered to avoid removing true variants.

Further, NGS incorporating UMIs or molecular barcodes [141,142] is a technique that has been adopted by several research groups for ctDNA profiling [126,139,143]. Similarly to their use in RNA sequencing (described above), the general concept is to use UMIs to uniquely label each DNA molecule prior to PCR amplification and library generation, as genuine mutations are expected to be present in all reads derived from a single DNA molecule and any differences between reads with a specific UMI would represent technical noise. NGS incorporating UMIs has increased the limit of detection to up to 1 in 10 million, but the proper design of UMIs is crucial to the success of such experiments and to avoid insufficient barcode diversity, thus falsely removing true variants. For a comprehensive review on improving NGS accuracy using UMIs, the readers are referred to Salk et al. [134].

Together, a number of computational methods for ctDNA assessment are evolving to increase the accuracy of NGS, and allowing increasingly accurate identification of cancer-relevant variants among a plethora of other changes.

## 6. Liquid Biopsy in Current Clinical Practice

Given its potential, over the last few years liquid biopsy has begun to attract interest in the context of routine molecular diagnostics.

While ctDNA analysis has been optimized for routine diagnostic use (i.e., with stringent standard operating procedures and good clinical practice compliance), for CTCs there is no such established practice yet, beyond enumeration [21], with most applications confined to research purposes. Thus, given the current limited use of CTCs in routine diagnostics, we will focus our attention on ctDNA-based procedures that are adopted in the clinic.

Liquid biopsy (restricted to ctDNA analysis) was officially adopted as a diagnostic tool in major reference center laboratories during the course of 2016. Internationally external quality assessment (EQA) round robin trials are currently available (German Society of Pathology, DGP) to assess laboratory performances in order to evaluate the accuracy of mutation testing across multiple centers. In any case, the reduced invasiveness of diagnostics-related procedures is a major advantage in a clinical routine setting (e.g., patients’ care benefits, reduction of medical complications that are associated with invasive procedures, and the possibility to achieve serial sampling), but also from a financial standpoint. For example, decreasing the instances of tissue biopsy collection could eventually lead to a reduction of hospitalization costs for the healthcare system (source: International Society for Pharmacoeconomics and Outcomes Research).

Despite its attractiveness, liquid biopsy testing is still limited to a restricted number of clinical applications, including primary tumor diagnosis, assessment of treatment response, therapy monitoring, detection of minimal residual disease, evaluation of tumor heterogeneity and emergence of resistances to targeted therapies. Currently, the determination of resistance to targeted therapy is the main driver for routine molecular diagnostics [144]. From a technical standpoint, blood collection is conducted in specific vessels coated with stabilizing agents that prevent contamination with genomic DNA originating from nucleated blood cell lysis [145]. The most commonly used solutions, largely implemented in clinical studies [146,147,148,149], are provided by Streck (Cell-free DNA BCT), Qiagen (PAXgene Blood ccfDNA Tubes) and Roche (CE-IVD Cell-free DNA Collection Tube). Ethylenediaminetetraacetic acid (EDTA) tubes, typically costing less than 1/10 of the aforementioned solutions, can be used as a valid alternative [150]. However, the processing of samples must occur within 2 to 4 h after phlebotomy for blood collected in EDTA tubes, compared to the one week of storage allowed in preservative-coated vessels [149,151,152]. Following plasma isolation, specific ctDNA extraction methods must be used to optimize the yield and preserve ctDNA integrity. Currently available kits are based on silica gel membrane columns, magnetic beads or polymer-mediated enrichment and are offered as fully automated or manual solutions. Several studies have compared the performances of these different extraction methods [153,154,155], resulting in comparable output with a similar price range. Upon extraction, the cfDNA amount is typically measured using intercalating fluorophores (PicoGreen or Qubit Fluorometer), yet a quality control step using capillary electrophoresis is recommend to evaluate the quality and purity of the extracted material. cfDNA is then ready to be analysed and screened for genetic variants using different technologies.

Despite Sanger sequencing still being considered the “gold standard” method for molecular analysis in tissue biopsies, technical limitations (e.g., a high detection limit) prevents the use of this technology for ctDNA analysis. Instead, other approaches such as dPCR, quantitative PCR (qPCR) and NGS are used to this end, with dPCR having the highest analytical sensitivity [156,157]. Nonetheless, the main constraint of dPCR resides in the limited number of targets that can be tested in each reaction (usually a restricted number of hotspots for one or two genes). In countries where stringent diagnostic regulations are in place (e.g., the USA), liquid biopsies are commonly analyzed using FDA-cleared qPCR assays, such as Therascreen (Qiagen) and Cobas (Roche) kits in CLIA-CAP accredited laboratories. This companion diagnostic test is used to assess the mutational status of *EGFR* exon 19 and exon 21 to identify patients with advanced non-small-cell lung cancer (NSCLC) that are eligible for treatment with EGFR tyrosine kinase inhibitors (EGFR-TKI). Currently, the analysis of tyrosine kinase inhibitor-resistance in NSCLC to assess the presence of *EGFR* T790M mutation is by far the most common application of ctDNA analysis in routine diagnostics worldwide, with the FDA and the European Medicines Agency (EMA)’s most recent recommendations suggesting the use of liquid biopsy testing in those cases where no tissue material is available [158]. Importantly, *EGFR* T790M plasma-positive patients treated with third generation EGFR-TKI osimertinib show an increased response rate and survival compared to tissue-positive but plasma-negative patients, indicating that in this case the molecular analysis of ctDNA is a stronger predictive factor compared to tissue [158,159]. However, despite being fully validated, these qPCR kits have a detection limit of up to >1% allelic frequency [160] and therefore are rarely used in European countries where LDTs (laboratory developed tests) with higher sensitivity are allowed.

By combining its multiplexing flexibility and adequate limit of detection, targeted NGS represents the most commonly used (although the most expensive) approach among the cited ones, with reagents for a targeted panel of up to ~50 genes typically costing between 350 and 450 USD per sample. The introduction of molecular barcodes has drastically improved the sensitivity of the amplicon sequencing methods (down to 0.1%), however this comes at additional cost along with the extremely high level of sequencing depth required (i.e., ~25,000× coverage) [139]. At last, while certainly representing an attractive option to comprehensively characterize the tumor molecular landscape, whole-exome sequencing (WES) is very rarely used for routine liquid biopsy testing. The main reason for this is the prohibitive cost for such an approach and the lack of designed kits for WES ctDNA testing, together with the (current) absence of a substantial clinical benefit deriving from WES analysis as opposed to targeted sequencing [160]. The vast majority of mutations detected by WES compared to targeted panels are commonly of unknown functional value and therefore not yet informative for instructing treatment decisions [160].

Even though mounting evidence for its clinical utility has been accumulated within recent years, liquid biopsy still finds limited space in current routine diagnostics, mostly confined to ctDNA analysis for resistance mutations. The implementation of internationally established guidelines, the execution of clinical studies, together with the improvement of standardization and harmonization across different platforms will eventually create higher confidence across the medical community to establish liquid biopsy as a widespread molecular diagnostic tool.

## 7. Conclusions and Future Perspectives

Liquid biopsy has recently emerged as a highly promising method to diagnose and monitor cancer. The analysis of CTCs and ctDNA currently appears to be complementary, with ctDNA providing a cost-effective and highly sensitive tool for the detection of mutations in a selection of cancer-associated hotspots, with CTCs representing a more labor-intensive yet highly promising tool to investigate drug sensitivity, tumor heterogeneity, mutations at a genome-wide scale, RNA and protein expression. It is likely that liquid biopsy will evolve in a cancer-specific fashion, focusing more on CTCs or ctDNA depending on the cancer type (or subtype) and the nature of its main alterations and mechanisms of drug resistance. Additionally, highly ambitious efforts are ongoing to use liquid biopsy for early cancer detection (i.e., before cancer-related symptoms occur), aiming towards an early diagnosis and possibly a better disease outcome [125]. More generally though, the implementation of liquid biopsy on a large scale in the clinical setting will require a better understanding of the full potential and limitations of this technology, which will be only possible to dissect with large patient cohorts in multiple cancer types and across multiple centers. Despite this, the high expectations for liquid biopsy as a novel tool for cancer diagnosis and monitoring are likely to boost research in this field in the years to come, with the objective of addressing many of the remaining questions and ultimately assessing whether liquid biopsy will be a breakthrough in the management of cancer patients.
